# Immune checkpoint inhibitor treatment of brain metastasis associated with a less invasive growth pattern, higher T-cell infiltration and raised tumor ADC on diffusion weighted MRI

**DOI:** 10.1007/s00262-023-03499-z

**Published:** 2023-07-21

**Authors:** Rasheed Zakaria, Michael D. Jenkinson, Mark Radon, Kumar Das, Harish Poptani, Nitika Rathi, Philip S. Rudland

**Affiliations:** 1 Department of Molecular and Clinical Cancer Medicine, University of Liverpool, Nuffield Building, Crown Street, Liverpool, L69 3BX UK; 2 Department of Pharmacology and Therapeutics, University of Liverpool, Liverpool, UK; 3 Department of Neuroradiology, Walton Centre NHS Foundation Trust, Liverpool, UK; 4 Department of Neuropathology, Walton Centre NHS Foundation Trust, Liverpool, UK

**Keywords:** Brain metastasis, Immunotherapy, Malignant melanoma, MRI, DWI, ADC

## Abstract

**Background:**

Brain metastases are the most common intracranial tumors with an increasing incidence. They are an important cause of morbidity and mortality in patients with solid organ cancer and a focus of recent clinical research and experimental interest. Immune checkpoint inhibitors are being increasingly used to treat solid organ cancers.

**Methods:**

To determine whether immune checkpoint inhibitors were biologically effective in the brain, we compared melanoma brain metastasis samples where treatment with ipilimumab had occurred preoperatively to those who had not received any immune modulating therapy and looked for histopathological (invasion, vascularity, metastasis inducing proteins, matrix metalloproteinases, immune cell infiltration, tissue architecture) and advanced MRI differences (diffusion weighted imaging).

**Results:**

Co-localized tissue samples from the same regions as MRI regions of interest showed significantly lower vascularity (density of CD34 + vessels) in the core and higher T-cell infiltration (CD3 + cells) in the leading edge for ipilimumab-treated brain metastasis samples than for untreated cases and this correlated with a higher tumor ADC signal at post-treatment/preoperative MRI brain.

**Conclusions:**

Treatment of a melanoma brain metastasis with ipilimumab appears to cause measurable biological changes in the tumor that can be correlated with post-treatment diffusion weighted MRI imaging, suggesting both a mechanism of action and a possible surrogate marker of efficacy.

**Supplementary Information:**

The online version contains supplementary material available at 10.1007/s00262-023-03499-z.

## Introduction

Brain metastases (BrM) are the most common intracranial tumors, showing increasing incidence due to improved detection and prolonged cancer survival, while the factors associated with outcomes are changing over time [1]. Immune checkpoint inhibitors (ICI) have intracranial efficacy in up to half of cases with metastatic melanoma in clinical trials [2]. It is increasingly understood that BrM may have different patterns of local invasion in vivo and in vitro, and this may relate to the immune microenvironment [3–5]. The effect of ICI on growth pattern in BrM is important, since local recurrence is a major problem after surgery and radiotherapy [6]. However, this question has not been addressed and only a small number of reports on the effect of ICI on BrM exist, none with imaging correlates [7].

It is unclear how best to monitor response to ICI in BrM because simple measures of size and edema may represent treatment effect rather than simply progression [8, 9]. Diffusion weighted imaging (DWI) is an advanced MRI sequence readily obtained in clinical practice that appears to be comparable across centers [10]. Apparent diffusion coefficient (ADC) is a crude surrogate marker of cellularity obtained from DWI sequences, with increased diffusion associated with reduced cellularity [9]. We performed a simple descriptive study on patients undergoing surgical resection of brain metastases from malignant melanoma. We used tissue and co-localized MRI studies including DWI to examine the histopathological and imaging features in patients with and without prior ICI and to look for any potential surrogate markers of efficacy that could be further investigated.

## Materials and methods

Four consecutive patients underwent resection of a melanoma BrM as part of routine clinical care. Ethical approval was from the institution’s tissue bank with patients’ written consent (NRES 20/WA/0043), and no additional surgical procedures or maneuvers aside from the standard of care *en bloc* tumor resection were performed [11]. Patients were all treated with dexamethasone 8 mg twice daily for at least 48 h prior to surgery which was tapered off post-operatively to clinical symptoms. Clinical details are given in Table 1, but all patients were of good performance status with a symptomatic solitary tumor deemed most suitable for neurosurgical removal at tumor board.

**Table 1:** Clinical characteristics of treated patients. Karnofsky performance status (KPS)

Age	Gender	KPS at surgery	Extracranial metastases at surgery	Controlled primary disease at surgery	Ipilimumab pre-surgery	Overall survival after surgery/months	Local progression free survival after surgery/months
35	F	90	Y	Y	N	5.2	3.5
53	M	90	Y	Y	N	3.6	3.3
64	F	80	N	Y	N	5.5	4.1
24	M	100	Y	Y	Y	4.9	2.9

### MRI studies and image analysis

Patients underwent a presurgical brain MRI < 14 days prior to operation that included DWI sequences and post-contrast T1W volume [12]. DWI with single-shot echo planar imaging at *b* values of 0 and 1000 s/mm^2^ was obtained with the following parameters: Achieva Philips 3.0 T scanner with proprietary head coil, spin echo “DwiSE” sequence, total acquisition time 44.7 min, repetition time/echo time 2828/73 ms, field of view 230 × 230 mm, acquired matrix 128 × 128, slice thickness 4 mm, 27 slice per volume, one volume without diffusion weighting (*b* = 0 s/mm2) followed by 32 diffusion sensitized images with gradients applied in non-collinear directions (*b* = 1000 s/mm2). For diagnosis and neuro-navigation, a volumetric fast spoiled gradient echo sequence was then taken after gadolinium injection at a standard dose of 0.1 mmol/kg (repetition time/echo time 9/1.4 ms, flip angle 15 degrees, acquisition matrix 256 × 256, volume 180 × 1 mm slices at zero angle gantry); this is referred to as the planning scan.

Raw DICOM data were imported into DTIstudio, version 3.0.3. Using the default settings (affine linear transformation, tri-linear interpolation, standard derivation of the ratio image), realignment and co-registration to b0 images were performed to remove eddy current distortions, and quality control of baseline images was also checked visually. An ADC trace map from DWI was then generated. Post-operatively, the vector specifying the biopsy location was extracted and transferred to image analysis software. A region of interest (ROI) 5 mm diameter (matching the size of the biopsy) was co-localized to the biopsy location. ADC measurements were recorded from each ROI matched to its tissue sample using a validated method [13]. Control measurements were taken from unaffected contralateral white matter and readings normalized to these values as is conventional in diffusion and perfusion MR studies.

### Tissue analysis

**Fig. 1:**
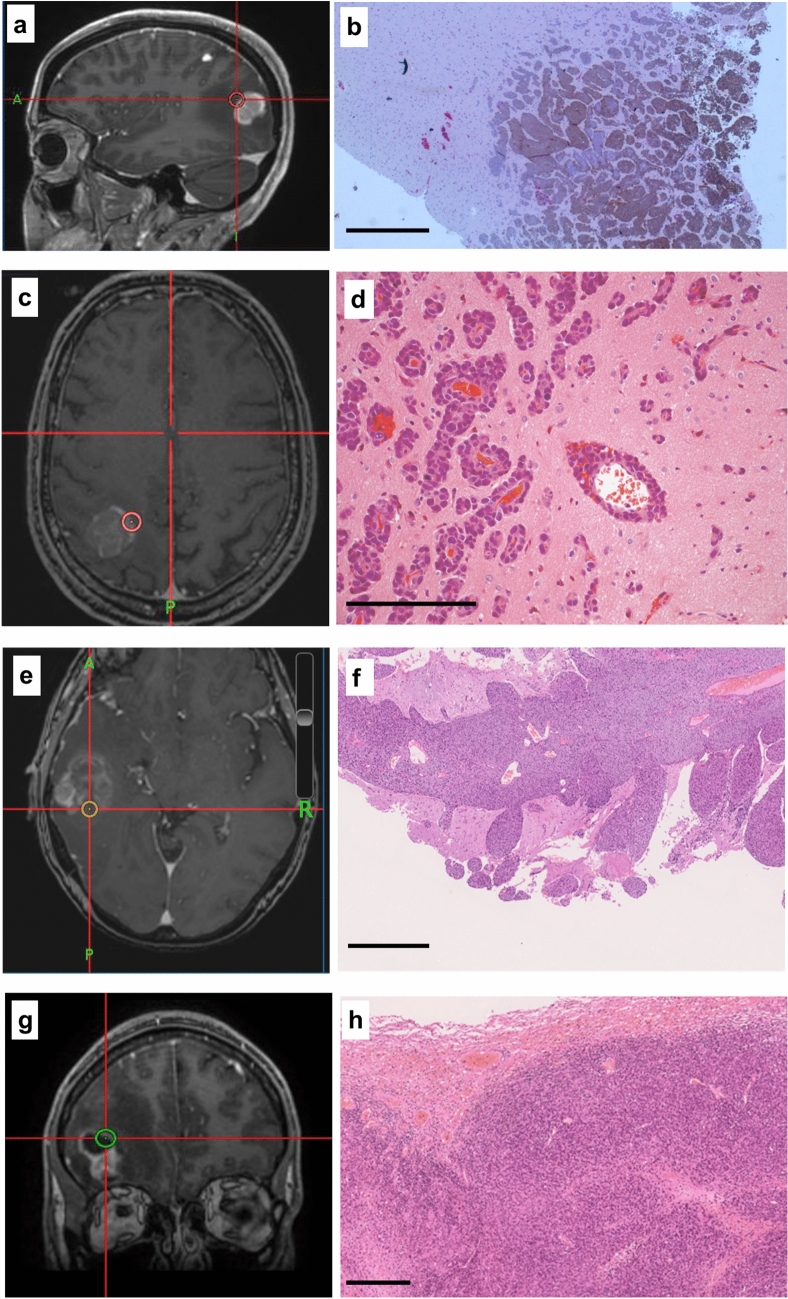
**a**, **c**, **e** left hand panels show T1W MRI brain scan (volume, post-contrast) from three patients with metastatic melanoma undergoing image-guided neurosurgical resection of a solitary brain metastasis and the tumor edge image-guided biopsy location (5 mm diameter). H&E-stained sections of the tissue specimens from the locations in the left hand panels illustrate tumor infiltration into surrounding white matter in **b**, **f** (scale bar = 1 mm) and perivascular growth, **d** (scale bar = 100 µm). A fourth patient had been treated with immune checkpoint inhibition prior to surgery—sample location in g—and showed a much more circumscribed border between tumor and white matter, **h** (H&E, scale bar = 1 mm). Post-operatively the structural MRI scans shown were fused to diffusion weighted scans and readings of ADC taken from the same ROI as the tumor/tissue specimen

**Fig. 2:**
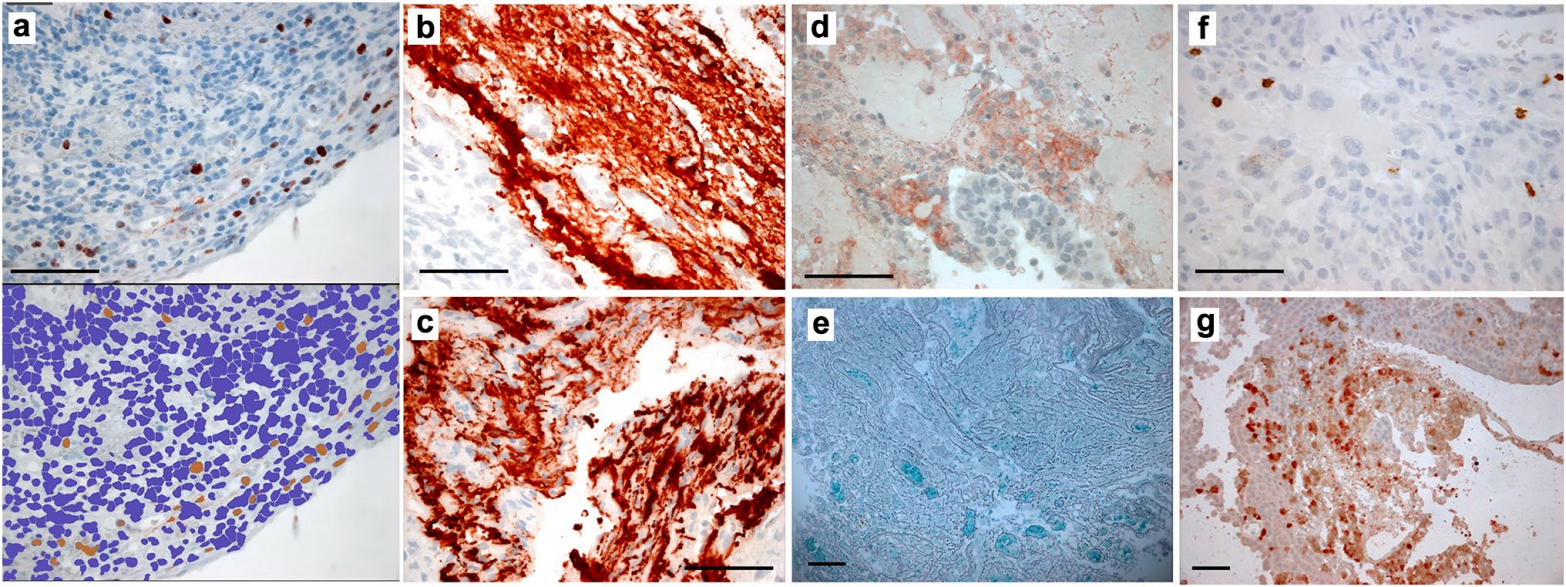
Histology and immunohistochemistry-stained sections of tumor specimens were assessed using previously described [3] methods for **a** proliferation (Ki67). The upper panel shows the raw stained slide and the lower panel the image generated by scanning the slide and using the automated plugins in ImageJ software—illustrated for the same sample from a patient treated with immune checkpoint inhibition (ICI) prior to surgery—the final percentage for this field was 2.3%, but all scores were checked manually (see Methods). GFAP staining in peritumoral samples from a control case **b** and ICI treated patient **c** density was assessed again using automated plugins and checked manually. The necrosis per high-powered field was assessed as a percentage scored by two observers and averaged for the core and edge specimens from each case, illustrated for a sample from one of the control cases in **d** with viable tumor stained with S100A4 and adjacent necrosis. Silver staining for reticulin was performed and scored for density as this has been shown to correlate with ADC in a prior study [5]—here in a peritumoral sample from the ICI treated case **e**. Immune cells were identified using standard antibodies for T-cells, B-cells and macrophages per the Methods and Supplementary Table and cells per high-powered field counted by two observers and checked by a third with good agreement, illustrated for CD8 in a control case in **f** and **g** ICI treated case. Magnification varies, scale bar = 100 µm throughout

During resection, image-guided tissue samples were taken using a standard neuronavigational platform in a method described previously but easily replicated [14].A neuropathologist categorized the growth pattern of all cases using tissue from the BrM margin blind to the other MRI and immunological stains, using a previously described system as either encapsulated, perivascular invasion or diffuse invasion [2]. Samples were marked with a dot of black medical ink in the operating room to show the deep pole, then stored in formalin for up to 24 h before processing and embedding in paraffin wax blocks with the orientation preserved. Histological sections were cut at 4 µm, transferred to APES-coated slides, dewaxed in xylene and rehydrated through graded ethanol to water. Stained slides (antibodies listed in Supplementary Table 1) were scored for markers of proliferation, metastasis, inflammation, connective tissue and vasculature using a well described semi-quantitative method for the percentage and intensity of staining by two separate observers [12]. Immune cells were counted per high-powered field (0.5 mm^2^ at 400 × magnification) in > 5 fields (average 6 per sample) in the core and the edge samples as well as in the peritumoral region (defined as < 1 high-powered field from the tumor boundary on the edge sample) and distant white matter (> 2 high-powered fields from tumor edge). Cell counts were highly consistent between observers, using intra-class correlation coefficient testing (Cronbach’s alpha = 0.762, *p* < 0.001). For Ki67, GFAP and connective tissue density, automated analysis with NIH ImageJ software was performed; slide photographs were taken using a Leica DFC310FX camera attached to a DM2000 microscope with the LAS V3 software suite (Leica microsystems, 2014) with no additional filtering and a Hamamatsu NanoZoomer S20MD slide scanner. Illustrations of the stained tissues are shown in Figs. 1 and 2.

## Results

**Fig. 3:**
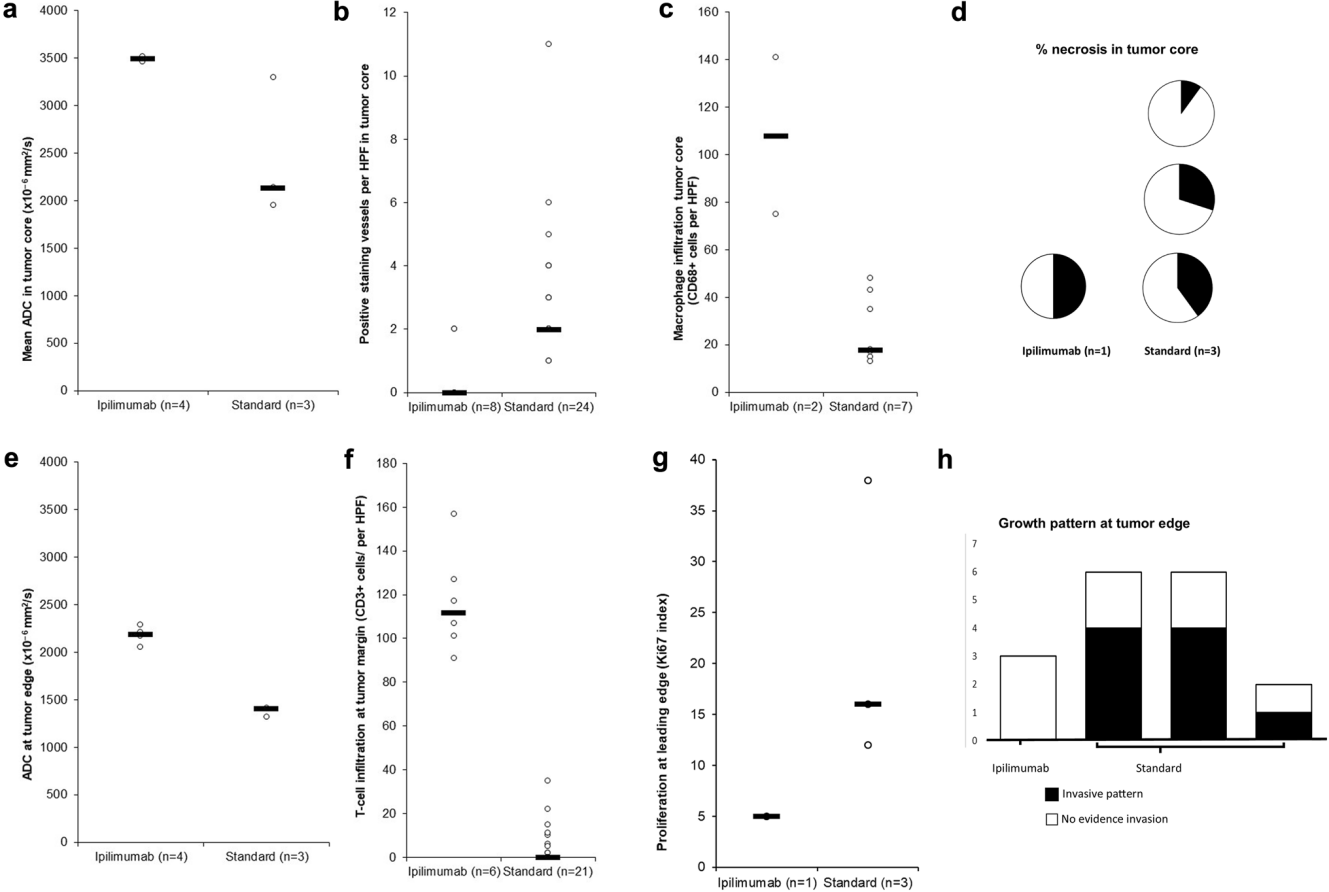
ADC readings from the ROIs of: **a** metastatic tumor core and **e** tumor leading edge. These readings were significantly higher in the patient treated before surgery with the immune checkpoint inhibitor ipilimumab (Mann–Whitney U tests *p* < 0.05). In the tumor core, the co-localized tissue samples for the ipilimumab-treated patient showed the higher ADC region had: **b** a significantly lower density of blood vessels, **c** significantly higher macrophage infiltration (Mann–Whitney U tests *p* < 0.05) and **d** a trend to higher % necrosis (*p* = 0.158), as scored by two independent observers. At the tumor leading edge, the regions with higher ADC were found to have: **f** significantly higher T-cell infiltration (Mann–Whitney U test *p* < 0.05), **g** a trend to lower cellular proliferation (% Ki67, *p* = 0.362) and **h** no evidence of tumor invasion into the adjacent white matter of the brain compared to controls not treated with ipilimumab (0/3 vs. 9/14 specimens, respectively; Fisher ET, *p* = 0.08) NB. n refers to number of samples/regions/high-powered fields (HPF) analyzed, and the number of tissue samples analyzed was not associated with an image-guided region of interest in a 1:1 fashion as multiple samples could be obtained from a single MRI region and multiple high-powered fields could be assessed in a single sample

Patients survived median 5.1 months (range 3.6–5.5) following operation, and there were no surgical complications. Three samples from one patient who had been treated with ipilimumab prior to surgery (ipi–BrM) showed a circumscribed growth pattern compared to an invasive growth pattern in 14 samples from three patients who had never received ICI (MBrM), shown in Fig. 1. There were no samples showing microscopic invasion in the ipi–BrM case compared to the MBrMs (0/3 vs. 9/14; Fisher ET, *p* = 0.08). There were no confounding differences between control and ipilimumab-treated cases for any of the other histological markers including the degree of edema as scored by a previously published scale [15]. The mean ADC at the tumor edge and within the tumor core was significantly higher in the ipi–BrM than the untreated MBrM (Mann–Whitney U, *p* < 0.05) as illustrated in Fig. 3. Co-localized tissue samples from the same regions showed significantly lower vascularity (density CD34 + vessels) in the core of the ipi–BrM than in the MBrM (Mann–Whitney U, *p* < 0.01), and these samples tended to have more necrosis (50% vs. 27%, *p* = 0.158) and lower proliferation (Ki67 score, 12% vs. 20%, *p* = 0.362) illustrated in Fig. 2. Higher T-cell infiltration (CD3 +) in leading edge and higher macrophage infiltration (CD68 +) in tumor core were seen in the ipi–BrM than in the untreated MBrM (Mann–Whitney U, *p* < 0.01), shown in Fig. 3.

## Discussion

It is unusual to obtain image-guided tissue samples from BrM patients prospectively; hence, the number of samples was small (only three in a single BrM treated with ICI then surgery) which limits the value of any conclusions. This is nonetheless a novel report of histological correlation with MRI changes after ICI showing that the co-localized tissues from an ipilimumab-treated BrM patient showed a greater immune cell infiltration and lower invasiveness compared to untreated controls from the same primary cancer type. It also demonstrates that as well as macrophage and T-cell infiltration, high-ADC readings from the same region at MRI correlated with lower vascularity and a trend to lower proliferation and higher necrosis. This is important as most BrM are treated without obtaining tissue, and therefore, non-invasive markers of microenvironmental change are urgently needed to guide treatment decisions. Diffusion is a crude measure of cellularity and tumor response; more sophisticated time dependent diffusion studies may allow more detailed assessment of what cell types are present including T-cells but this at the preclinical stage [16]. Perfusion MRI would have been extremely valuable to assess the correlation with immune infiltration and vessel density, and this would be a logical next step to look for further imaging biomarkers of treatment response. Future studies on the effects and timing of immunotherapy and surgery on one another for BrM are indicated as this is a small comparison of only one treated case and three controls which generates rather proves any hypotheses [17, 18].

## Supplementary Information

Below is the link to the electronic supplementary material.

Supplementary file1 (DOCX 30 KB)
