# Supplementary material for: Immune checkpoint inhibitor treatment of brain metastasis associated with a less invasive growth pattern, higher T-cell infiltration and raised tumor ADC on diffusion weighted MRI
Source: Cancer Immunol Immunother. 2023 Jul 21;72(10):3387–93. doi: 10.1007/s00262-023-03499-z (PMC10491542; doi:10.1007/s00262-023-03499-z)
Supplement: Supplementary file 1 — Supplementary file1 (DOCX 30 KB) [file 262_2023_3499_MOESM1_ESM.docx]

**Supplementary methods**

| Marker | Function | Antibody / stain | Dilution | Antigen retrieval |
| --- | --- | --- | --- | --- |
| CD34 | Endothelial antibody used to assess vascularity (1) | Mouse monoclonal #M7165  Dako | 1:25  EnVmse | Proteinase K |
| GFAP | Filament protein found in astrocytes and used to assess reactive gliosis (2) | Rabbit polyclonal  #Z0334  Dako | 1:1500  EnVrbt | Proteinase K |
| CD20 | B cell differentiation antigen (3) | Mouse monoclonal  #M0755  Dako | 1:300  EnVmse | Citrate buffer (pH6) |
| CD68 | Receptor involved in phagocytosis and expressed on macrophages (4) | Mouse monoclonal  #M0814  Dako | 1:100  EnVmse | Citrate buffer (pH6) |
| CD3 | Non-specific T cell marker (5) | Rabbit polyclonal  #A0452  Dako | 1:50  EnVrbt | Tris-EDTA buffer (pH9) |
| Reticulin | Component of extra-cellular matrix (6) | Silver stain(7) |  |  |
| S100A4 | Calcium binding protein shown to promote metastasis in several animal models (8); overexpression associated with intracranial progression in brain metastases(9) | Rabbit polyclonal  #A5114  Dako | 1:400  EnVrbt | none |
| OPN | Varied role in cell-matrix interaction, binds the cell surface integrins α_V_β_3_/α_V_β_5_ and promotes metastasis (10-12). May be involved in radiation response in human brain metastases.(9) | Mouse monoclonal  #MP111B10(1)  Developmental Studies Hybridoma Bank(DSHB) | 1:300  EnVmse | none |
| S100P | Calcium binding protein inducing metastasis in breast cancer animal models (13). | Mouse monoclonal  #610307  BD Biosciences | 1:75  EnVmse | none |
| AGR2 | Normal component of ER highly overexpressed in range of human cancers. (14). | Rabbit polyclonal  #ARP42290  Aviva Systems Biology | 1:750  EnVrbt | none |
| MMP2 | Matrix metalloproteinases are zinc- dependent, secreted endopeptidases essential for degradation and remodelling of the extracellular matrix. They are highly implicated in metastasis to and survival of cancer cells in the brain.(15, 16) | Mouse monoclonal  #MAB13431  Chemicon | 1:40  EnVmse | none |
| MMP9 |  | Rabbit monoclonal  #ab137867  Abcam | 1:100  EnVrbt | none |
| MMP13 |  | Mouse monoclonal  #MA5-14238  Thermo Scientific | 1:60  EnVmse | none |
| Ki67 | Marker of cell proliferation(17) | Mouse monoclonal  #M7240  Dako | 1:75  EnVmse | Tris-EDTA buffer (pH9) |

**References**

1. Ramani P, Bradley NJ, Fletcher CD (1990) QBEND/10, a new monoclonal antibody to endothelium: assessment of its diagnostic utility in paraffin sections. Histopathology. 17: 237-42.

2. Frank E, Pulver M, de Tribolet N (1986) Expression of class II major histocompatibility antigens on reactive astrocytes and endothelial cells within the gliosis surrounding metastases and abscesses. J Neuroimmunol. 12: 29-36.

3. Reinherz EL, Haynes BF, Nadler LM, Bernstien ID, SpringerLink (Online service) (1986) Leukocyte Typing II Volume 2 Human B Lymphocytes. Springer New York,, New York, NY. pp. 1 online resource.

4. Micklem K, Rigney E, Cordell J, Simmons D, Stross P, Turley H, Seed B, Mason D (1989) A human macrophage-associated antigen (CD68) detected by six different monoclonal antibodies. Br J Haematol. 73: 6-11.

5. Reinherz EL, Haynes BF, Nadler LM, Bernstein ID, SpringerLink (Online service) (1986) Leukocyte Typing II Volume 1: Human T Lymphocytes. Springer New York,, New York, NY. pp. 1 online resource.

6. Bolonyi F (1958) Study of the reticulin fibers of brain tumors. J Neuropathol Exp Neurol. 17: 240-6.

7. Gordon H, Sweets HH (1936) A simple method for the silver impregnation of reticulum. The American journal of pathology. 12: 545-52.1.

8. Gross SR, Sin CG, Barraclough R, Rudland PS (2014) Joining S100 proteins and migration: for better or for worse, in sickness and in health. Cellular and molecular life sciences : CMLS. 71: 1551-79. doi: 10.1007/s00018-013-1400-7

9. Zakaria R, Platt-Higgins A, Rathi N, Crooks D, Brodbelt A, Chavredakis E, Lawson D, Jenkinson MD, Rudland PS (2016) Metastasis-inducing proteins are widely expressed in human brain metastases and associated with intracranial progression and radiation response. British journal of cancer. 114: 1101-8. doi: 10.1038/bjc.2016.103

10. Anborgh PH, Mutrie JC, Tuck AB, Chambers AF (2010) Role of the metastasis-promoting protein osteopontin in the tumour microenvironment. Journal of cellular and molecular medicine. 14: 2037-44. doi: 10.1111/j.1582-4934.2010.01115.x

11. Hahnel A, Wichmann H, Kappler M, Kotzsch M, Vordermark D, Taubert H, Bache M (2010) Effects of osteopontin inhibition on radiosensitivity of MDA-MB-231 breast cancer cells. Radiation oncology (London, England). 5: 82. doi: 10.1186/1748-717x-5-82

12. Guttler A, Giebler M, Cuno P et al. (2013) Osteopontin and splice variant expression level in human malignant glioma: radiobiologic effects and prognosis after radiotherapy. Radiotherapy and oncology : journal of the European Society for Therapeutic Radiology and Oncology. 108: 535-40. doi: 10.1016/j.radonc.2013.06.036

13. Wang G, Platt-Higgins A, Carroll J, de Silva Rudland S, Winstanley J, Barraclough R, Rudland PS (2006) Induction of metastasis by S100P in a rat mammary model and its association with poor survival of breast cancer patients. Cancer research. 66: 1199-207. doi: 10.1158/0008-5472.can-05-2605

14. Barraclough DL, Platt-Higgins A, de Silva Rudland S, Barraclough R, Winstanley J, West CR, Rudland PS (2009) The metastasis-associated anterior gradient 2 protein is correlated with poor survival of breast cancer patients. The American journal of pathology. 175: 1848-57. doi: 10.2353/ajpath.2009.090246

15. Vandenbroucke RE, Libert C (2014) Is there new hope for therapeutic matrix metalloproteinase inhibition? Nat Rev Drug Discov. 13: 904-27. doi: 10.1038/nrd4390

<http://www.nature.com/nrd/journal/v13/n12/abs/nrd4390.html#supplementary-information>

16. Cathcart J, Pulkoski-Gross A, Cao J (2015) Targeting matrix metalloproteinases in cancer: Bringing new life to old ideas. Genes & Diseases. 2: 26-34. doi: 10.1016/j.gendis.2014.12.002

17. Plate KH, Ruschoff J, Behnke J, Mennel HD (1990) Proliferative potential of human brain tumours as assessed by nucleolar organizer regions (AgNORs) and Ki67-immunoreactivity. Acta Neurochir (Wien). 104: 103-9.
